# Potential Prognostic and Predictive Role of Monocyte and Lymphocyte Counts on Presentation in Patients Diagnosed With Diffuse Large B-Cell Lymphoma

**DOI:** 10.7759/cureus.35654

**Published:** 2023-03-01

**Authors:** Darine M Kharroubi, Ghazi Nsouli, Zeinab Haroun

**Affiliations:** 1 Clinical Pathology, Rafic Hariri University Hospital, Beirut, LBN; 2 Oncology, Rafic Hariri University Hospital, Beirut, LBN; 3 Obstetrics and Gynecology, Rafic Hariri University Hospital, Beirut, LBN

**Keywords:** diffuse large b-cell lymphoma, progression-free survival, overall survival, prognosis, lymphocyte-to-monocyte ratio

## Abstract

Introduction: Diffuse large B-cell lymphoma (DLBCL) is the most common subtype of non-Hodgkin’s lymphoma. Nearly 40% of patients will die of relapsed disease despite the use of rituximab plus cyclophosphamide, doxorubicin, vincristine, and prednisone (R-CHOP) chemotherapy. Many prognostic markers established in the chemotherapy era are no longer valid in the rituximab era.

Objectives: We aim to identify whether we can add absolute lymphocyte count (ALC), absolute monocyte count (AMC), and lymphocyte-to-monocyte ratio (LMR) as new prognostic factors for DLBCL treated with R-CHOP. We also aim to find whether a correlation exists between these variables and the revised International Prognostic Index (R-IPI) score.

Methods: This is an observational retrospective study done from 2005 to 2015 in Rafic Hariri University Hospital (RHUH), Lebanon, on 42 patients treated with R-CHOP. Patients’ data were obtained from medical records. We used receiver operating characteristic (ROC) curve for establishing cutoff values. The chi-square test was used to analyze associations between variables.

Results: Patients were followed for a median of 42 months (24-96 months). Patients with LMR < 2.53 had a significantly worse outcome than those with LMR ≥ 2.53 (*p *< 0.0001). This was also true for patients with ALC < 1.47 × 10^9^/L (*p *= 0.0163) and AMC > 0.603 × 10^9^/L (*p *= 0.0053). LMR was also able to risk-stratify patients within each R-IPI category into high- and low-risk patients.

Conclusion: ALC, AMC, and LMR, surrogate markers of the host immune system and tumor microenvironment, have prognostic significance in DLBCL patients treated with R-CHOP.

## Introduction

Malignant lymphomas originate from cells of the lymphoid lineage at various stages of differentiation. The current WHO classification includes more than 70 different variants of lymphoid malignancies [1]. Diffuse large B-cell lymphoma (DLBCL), the most common malignant lymphoma in adults, is an aggressive lymphoma with variations in clinical behavior. Major efforts have been made to better understand the biology of DLBCL for better predicting the prognosis and response to treatment. DLBCL accounts for 30%-40% of non-Hodgkin’s lymphoma (NHL) cases [2]. The CHOP (cyclophosphamide, doxorubicin, vincristine, and prednisone) chemotherapy regimen has been the mainstay of treatment. The addition of the anti-CD20 monoclonal antibody rituximab to this chemotherapy significantly improved outcomes, and R-CHOP was established as the standard of care. About 60% of patients will be cured, with limited-stage disease patients showing better outcomes. Despite these major changes upon DLBCL frontline treatment, 10%-15% of patients exhibit primary refractory disease (no response or relapse within three months of therapy) and 20%-25% relapse after initial response to therapy, with most relapses occurring within two years of treatment. More than half of patients with DLBCL respond to R-CHOP, but the rest will usually die because of early treatment failure, partial response, or relapse [3].

Lymphoma is not just a malignant mass of lymphocytes but has a complex structure, to which many other cells are recruited and can be altered by the transformed cells. The tumour microenvironment is defined by interactions between malignant and non-transformed cells. The non-malignant cells of the tumor microenvironment, including cells of the immune system, tumor vasculature and lymphatics, fibroblasts, and sometimes adipocytes, have a dynamic function at all stages of tumorigenesis [4]. There are many T-cell populations within the tumor microenvironment. Among these, memory cytotoxic CD8+ T cells are capable of killing the lymphoma cells and are strongly associated with a favorable prognosis. CD4+ T-helper 1 (TH1) cells, by producing interleukin-2 (IL-2) and interferon gamma (IFN-γ), support the CD8+ lymphocytes. High numbers of CD4+ TH1 in the tumor microenvironment are also correlated with a favorable prognosis. T-regulatory cells (T regs) can also suppress the tumor in some B-cell lymphomas, and their presence in Hodgkin’s lymphoma correlates with a favorable prognosis, most probably via direct suppression of tumor growth [5,6]. On the other hand, tumor-associated macrophages (TAMs) are abundant in the tumor microenvironment, and their activity is pro-tumorigenic. TAMs stimulate malignant cell invasion and metastases. Macrophages also play a role in enhancing tumor angiogenesis. There is evidence that an increased number of TAMs in the tumor is associated with a poor prognosis [7,8].

The International Prognostic Index (IPI), which was developed prior to the introduction of rituximab into the frontline treatment of DLBCL, is the main clinical prognostic factor predicting survival in patients. The IPI has five clinical characteristics presenting at the time of diagnosis. These characteristics are the age, stage, serum lactate dehydrogenase (LDH) level, Eastern Cooperative Oncology Group (ECOG) performance status, and extranodal site of disease. IPI categorizes patients into four groups with five-year overall survival (OS) from 26% to 73%. These groups are the low-risk group (score 0-1), low-intermediate group (score 2), high-intermediate group (score 3), and high-risk group (score 4-5). A retrospective analysis was done by Sehn et al. on 365 patients with DLBCL treated with R-CHOP in the province of British Columbia to explore the utility of the IPI score. IPI risk factors in this study were regrouped into a different classification with three different prognostic groups to form the revised IPI (R-IPI) with better outcome prediction. The risk factors for which one point was added to the score are age greater than 60 years, stage III/IV disease according to the Ann Arbor staging system, elevated serum LDH, ECOG performance status 3-4, and more than one extranodal site of disease. The “very good” prognostic group has zero risk factors with more than a 90% chance of long-term survival. The “good” prognostic group has one or two risk factors with about an 80% chance of long-term survival [9]. Finally, the “poor” risk group has three to five risk factors with about a 50% chance of long-term survival. This redistribution of IPI factors has shown that R-IPI is able to predict patients’ outcome better than IPI score. However, IPI and R-IPI do not predict those patients with less than a 50% chance of cure, who are most in need of a different therapeutic approach [10].

Some biomarkers that were previously identified in DLBCL patients appear to be no longer significant. Bcl-2 overexpression is no longer a negative prognostic factor after the addition of rituximab to CHOP regimen [11]. In addition, Bcl-6 protein expression is no longer a favorable prognostic factor in patients treated with immunochemotherapy since Bcl-6(+) cases did not benefit from the addition of rituximab to chemotherapy [12]. Attempts were also made in order to analyze any new prognostic factors affecting survival and capable of identifying high-risk population. Gene expression profiling (GEP) provides important information about the heterogeneity of tumor cells and allows molecular classification of DLBCL. Unfortunately, gene expression arrays are not always available, need fresh specimens, and are expensive. Immunohistochemical algorithms were proposed as an alternative to GEP, but they have limited reproducibility and are not prognostic in the rituximab era [13]. Thus, the IPI or R-IPI is now the current standard prognostic index, even after the introduction of rituximab.

Our aim is to study the possible role of absolute lymphocyte count (ALC) and absolute monocyte count (AMC) at admission as prognostic and predictive markers in patients with DLBCL treated with rituximab-based chemotherapy and whether there may be any possible correlation between these markers and R-IPI criteria. Inclusion of additional prognostic factors may allow better identification of patients who will suffer from an aggressive course of the disease, thus optimizing patient management.

## Materials and methods

 Study design

 This was an observational retrospective analysis of data of DLBCL patients diagnosed between 2005 and 2015 at Rafic Hariri University Hospital (RHUH), Lebanon.

 Study population

 *Patients*

 We reviewed 57 DLBCL cases admitted and treated between 2005 and 2015 in RHUH. A total of five patients were treated with chemotherapy alone due to the non-availability of rituximab. Eight patients did not return for follow-up after the last R-CHOP cycle and two were excluded by two exclusion criteria for our study (HIV-related lymphoma and CNS involvement). Patients were selected with the diagnosis of DLBCL confirmed by histology. All of them received R-CHOP intravenous (IV) chemotherapy, including rituximab (375 mg/m^2^ IV), cyclophosphamide (750 mg/m^2^ IV), doxorubicin (50 mg/m^2^ IV), vincristine (1.4 mg/m^2^ IV), and oral prednisone (40 mg/m^2^). We ended up with a total of 42 patients included in the study.

 *Inclusion Criteria*

 The inclusion criteria were the following: (1) histologically confirmed DLBCL; (2) adequate renal function as documented by serum creatinine level <2 x upper limit of normal (ULN); (3) adequate hepatic function as documented by total bilirubin ≤1.5 x ULN and alanine aminotransferase (ALT) and aspartate aminotransferase (AST) ≤2 x ULN; (4) ECOG performance status 0-2 (score 0: fully active, able to continue on all pre-disease performance without restriction; score 1: restricted in physically strenuous activity but ambulatory and able to perform light work; score 2: ambulatory >50% of the day and capable of self-care but cannot perform work activities); and (5) age ≥18 years old.

 *Exclusion Criteria*

 The exclusion criteria were as follows: (1) primary CNS lymphoma; (2) active CNS involvement; (3) HIV-related lymphoma; (4) ECOG performance 3-4 (score 3: capable of limited self-care only and ambulatory for <50% of the day; score 4: disabled, cannot perform any self-care, and totally confined to bed/chair); and (5) severe co-morbidities (including uncontrolled diabetes mellitus, clinically significant cardiovascular disease, and history of stroke or intracranial bleeding).

Methods of data collection

The data were collected from the patient’s medical records after getting approval from the Rafic Hariri University Hospital Institutional Review Board (IRB) committee. Patient characterization was done according to age, gender, ECOG performance status, serum LDH level, Ann Arbor stage, and number of extranodal sites involved, which was determined by bone marrow biopsy and computed tomography (CT) scan. Since R-IPI was demonstrated to be a better prognostic index than IPI, we calculated R-IPI score for the patients we studied. The ALC and the AMC were obtained by an automated complete blood counter at 8-9 a.m. for all patients. The lymphocyte-to-monocyte ratio (LMR) was calculated for each patient by dividing the ALC by the AMC. 

Patients with primary refractory disease (no response or relapse within three months of therapy), partial response, or relapse following initial response to R-CHOP or death due to disease progression were considered having a poor outcome. Patients with complete response and no relapse during follow-up were considered having a good outcome. 

Statistical analysis

 The statistical analysis was performed using GraphPad Prism. The chi-square test (X^2^ test) of independence and Fisher’s exact test of independence were used to determine if there is a significant relationship between two nominal (categorical) variables. The strength of the association was estimated using Cramer’s V. Cutoff optimization was performed using the software package Cutoff Finder [14]. Based on ROC curve, two methods (Manhattan and Euclidean distance) were used to calculate the optimal cutoffs by minimizing the distance on the ROC curve to the left top edge of the diagram. *p*-values <0.05 were considered significant.

## Results

Patients' characteristics

 The baseline patients’ characteristics are summarized in Table 1 and Table 2.

**Table 1:** Baseline patients’ characteristics. Baseline patients’ characteristics of our population including gender, ALC, AMC, LMR before initiating treatment, R-IPI score, number of R-CHOP cycles received by each patient, and response to R-CHOP (outcome). ALC: absolute lymphocyte count; AMC: absolute monocyte count; LMR: lymphocyte-to-monocyte ratio; R-IPI: revised International Prognostic Index; R-CHOP: rituximab plus cyclophosphamide, doxorubicin, vincristine, and prednisone; V.good: very good.

Gender	ALC (×10^9^/L)	AMC (×10^9^/L)	ALC/AMC	R-IPI score	R-CHOP cycles	Response
Male	2.3912	0.5673	4.22	0 V.good	6 R-CHOP	Good
Female	1.9782	0.8442	2.34	0 V.good	6 R-CHOP	Poor
Male	2.875	1.087	2.64	2 Good	12 R-CHOP	Good
Male	0.5782	0.5074	1.14	3 Poor	6 R-CHOP	Poor
Male	5.4312	0.9052	6.00	2 Good	15 R-CHOP	Good
Female	1.5387	0.483	3.19	1 Good	6 R-CHOP	Good
Female	1.5552	0.388	4.00	2 Good	6 R-CHOP	Good
Female	0.8554	1.0152	0.84	1 Good	6 R-CHOP	Poor
Male	2.1329	0.5544	3.85	3 Poor	8 R-CHOP	Good
Female	1.495	0.312	4.79	3 Poor	8 R-CHOP	Good
Male	2.7715	0.598	4.63	1 Good	5 R-CHOP	Good
Female	1.106	0.6873	1.61	0 V.good	8 R-CHOP	Good
Male	2.5536	0.5472	4.67	3 Poor	6 R-CHOP	Poor
Female	2.2401	0.5764	3.89	3 Poor	6 R-CHOP	Good
Female	1.836	0.714	2.57	1 Good	6 R-CHOP	Good
Female	1.625	0.5135	3.16	2 Good	8 R-CHOP	Good
Male	1.4529	0.609	2.39	4 Poor	6 R-CHOP	Poor
Female	2.618	0.952	2.75	0 V.good	6 R-CHOP	Good
Female	2.178	0.99	2.20	3 Poor	6 R-CHOP	Poor
Female	0.9984	1.0296	0.97	1 Good	6 R-CHOP	Poor
Male	1.827	1.0875	1.68	4 Poor	8 R-CHOP	Poor
Male	1.2994	0.6132	2.12	3 Poor	6 R-CHOP	Poor
Male	2.914	0.589	4.95	0 V.good	5 R-CHOP	Good
Female	0.9176	0.3689	2.49	3 Poor	6 R-CHOP	Poor
Female	1.062	0.4995	2.13	3 Poor	8 R-CHOP	Poor
Male	1.551	0.627	2.47	1 Good	6 R-CHOP	Poor
Female	2.3014	0.399	5.76	0 V.good	5 R-CHOP	Good
Female	1.248	0.2592	4.81	3 Poor	6 R-CHOP	Good
Male	1.6891	1.231	1.37	2 Good	8 R-CHOP	Poor
Male	2.7715	0.598	4.63	1 Good	6 R-CHOP	Good
Female	1.74	0.174	10.00	2 Good	5 R-CHOP	Good
Male	1.395	0.8649	1.61	3 Poor	7 R-CHOP	Poor
Male	0.8288	0.8029	1.03	1 Good	8 R-CHOP	Good
Male	0.5452	0.2842	1.92	2 Good	8 R-CHOP	Poor
Female	1.184	0.4096	2.89	0 V.good	6 R-CHOP	Good
Male	2.716	0.291	9.33	0 V.good	6 R-CHOP	Good
Male	2.178	0.891	2.44	3 Poor	6 R-CHOP	Poor
Female	2.195	1.232	1.78	1 Good	8 R-CHOP	Poor
Female	1.2168	0.7696	1.58	1 Good	6 R-CHOP	Poor
Male	0.909	0.303	3.00	3 Poor	8 R-CHOP	Good
Female	2.1252	0.805	2.64	2 Good	7 R-CHOP	Good
Male	1.3034	1.1613	1.12	2 Good	6 R-CHOP	Poor

**Table 2:** Other baseline patients’ characteristics. Distribution of patients according to gender, R-IPI score, and outcome. R-IPI: revised International Prognostic Index.

		Number of patients	Percentage
Gender	Men	21	50
	Women	21	50
R-IPI score	0 (very good)	8	19
	1;2 (good)	19	45.23
	3;4 (poor)	15	35.71
Outcome	Good	23	55
	Poor	19	45

The median age of all patients at diagnosis was 49 years (range of 18-86 years). Patients were equally distributed according to gender. Based on the R-IPI score, eight patients (19%) had R-IPI score 0 (very good prognosis), 19 patients (45%) had R-IPI score 1-2 (good prognosis), and the remaining 15 patients (36%) had R-IPI score 3-4 (poor prognosis). Patients were followed for a median of 42 months (range 24-96 months), and those with primary refractory disease (no response or relapse within three months of therapy), partial response, or relapse following initial response to R-CHOP or death due to disease progression were considered having a poor outcome (19 patients, 45%). Patients with complete response and no relapse during follow-up were considered having a good outcome (23 patients, 55%). The median follow-up for this group of patients having a good outcome was 45 months (range 24-96 months). The median ALC of all patients was 1.65 × 109/L (range 0.54 × 109/L to 5.43 × 109/L). The median AMC of all patients was 0.603 × 109/L (range 0.174 × 109/L to 1.232 × 109/L). The median LMR was 2.605 (range 0.84-10) (Table 3).

**Table 3:** Statistical parameters of ALC, AMC, and LMR. SD, standard deviation; CI, confidence interval; ALC: absolute lymphocyte count; AMC: absolute monocyte count; LMR: lymphocyte-to-monocyte ratio.

	ALC × 10^9^/L	AMC × 10^9^/L	LMR
Median (range)	1.657 (0.5452-5.431)	0.6035 (0.174-1.232)	2.605 (0.84-10)
Mean ± SD	1.813 ± 0.8738	0.6701 ± 0.2855	3.181 ± 1.998
95% CI of mean	[1.54 ; 2.085]	[0.5811 ;0.7591]	[2.558; 3.804]

Cutoff values of ALC, AMC, and LMR

**Figure 1:**
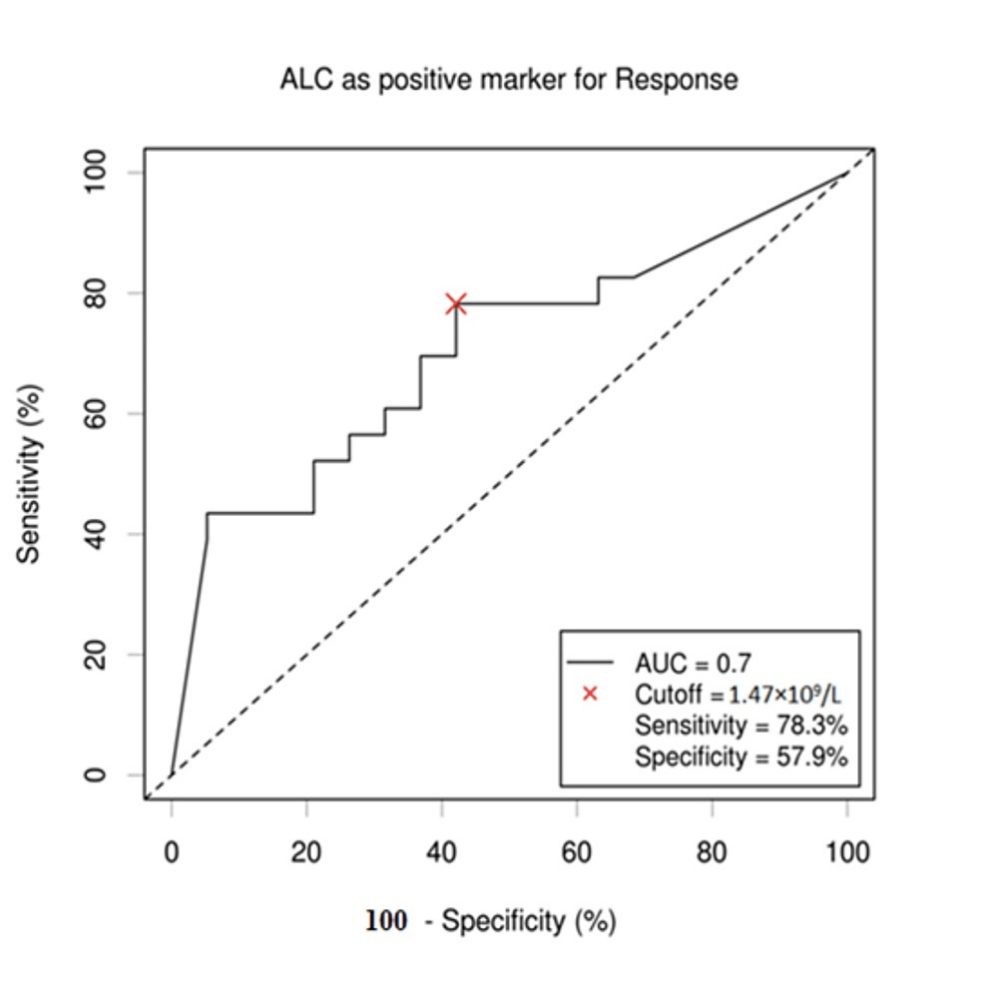
ROC curve of ALC value. When the cutoff value of ALC was 1.47 x 109/L, the sensitivity and specificity were 78.3% and 57.9%, respectively. The AUC value was 0.7 with 95% CI (0.565; 0.872). ALC: absolute lymphocyte count; ROC: receiver operating characteristic; AUC: area under the curve; CI: confidence interval.

**Figure 2:**
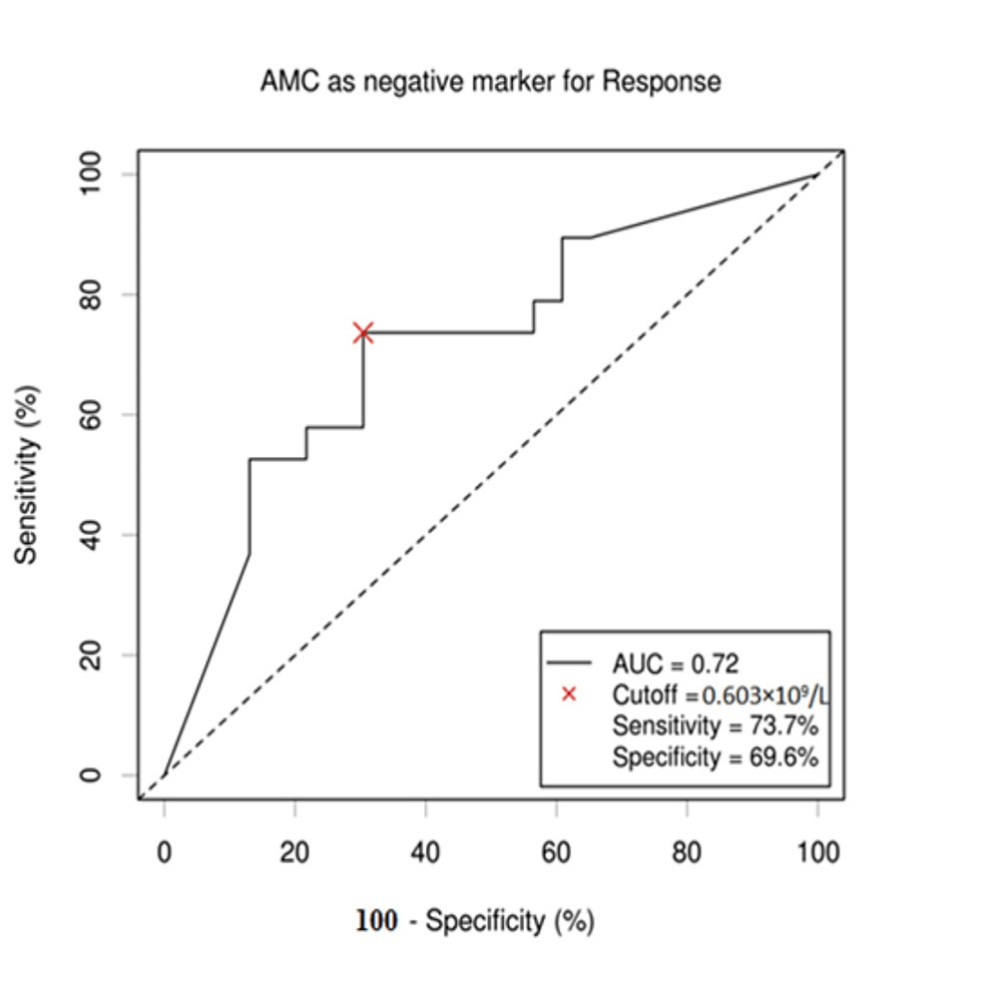
ROC curve of AMC value. When the cutoff value of AMC was 0.603 x 109/L, the sensitivity and specificity were 73.7% and 69.6%, respectively. The AUC value was 0.72 with 95% CI (0.624; 0.8126). AMC: absolute monocyte count; ROC: receiver operating characteristic; AUC: area under the curve; CI: confidence interval.

**Figure 3:**
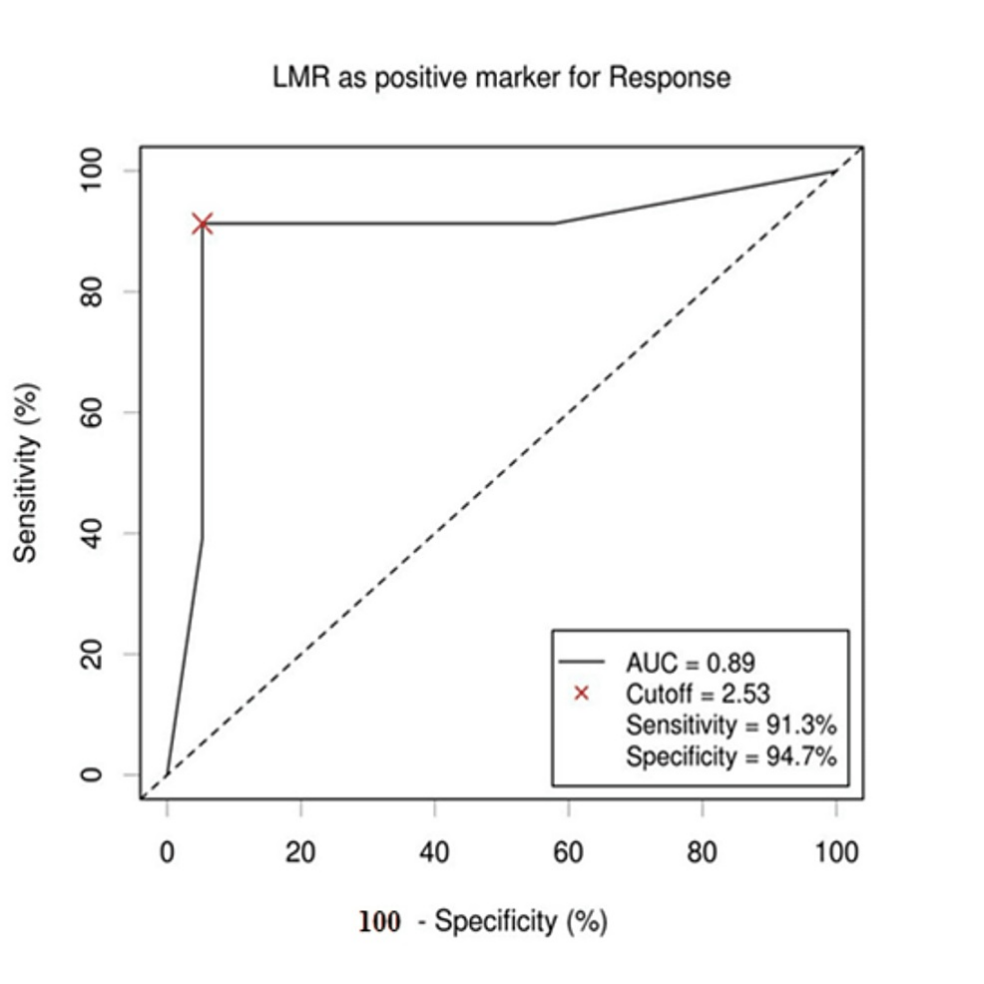
ROC curve of LMR value. When cutoff value of LMR was 2.53, the sensitivity and specificity were 91.3% and 94.7%, respectively. The AUC value was 0.89 with 95% CI (0.785; 0.995). LMR: lymphocyte-to-monocyte ratio; ROC: receiver operating characteristic; AUC: area under the curve; CI: confidence interval.

The cutoff values of AMC, ALC, and LMR for survival outcomes were selected by the ROC curve analysis. The cutoff value of ALC was established to be 1.47 × 109/L, with an area under the curve (AUC) value of 0.7 (95% confidence interval (CI): 0.565-0.872). This cutoff value showed maximum sensitivity and specificity on the ROC curve (78.3% sensitivity and 57.9% specificity). The cutoff value of AMC for outcome was established to be 0.603 × 109/L, with an AUC value of 0.72 (95% CI: 0.624-0.8126). This cutoff value showed maximum sensitivity and specificity on the ROC curve (73.7% sensitivity and 69.6% specificity). The LMR cutoff point for patients' outcome was established to be 2.53 with an AUC of 0.89 (95% CI: 0.785; 0.995). This cutoff correlated with the maximum sensitivity and specificity on the ROC curve (91.3% sensitivity and 94.7% specificity) (Figures 1-3).

Prognostic significance of ALC, AMC, and LMR

**Figure 4:**
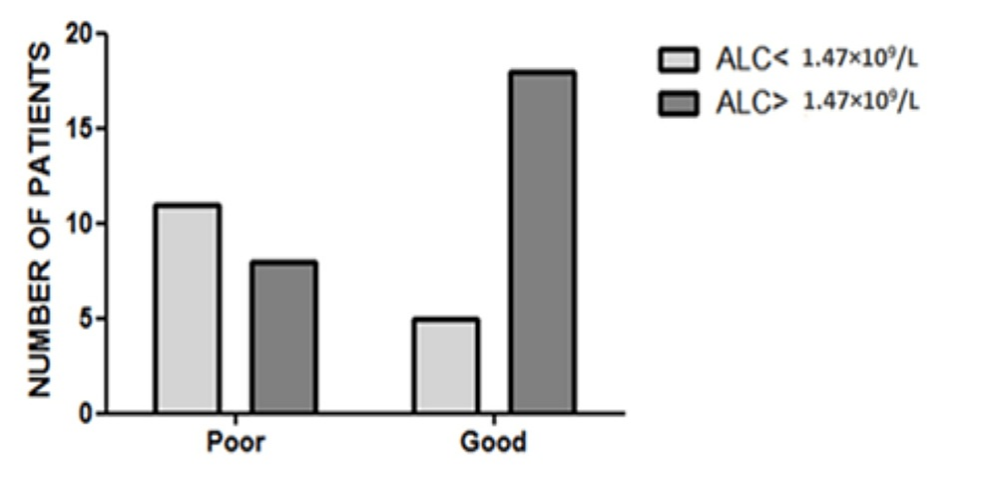
Patients’ distribution according to ALC cutoff and outcome. Significant moderate association between ALC and prognosis (*p *= 0.0163, Cramer’s V = 0.371). ALC: absolute lymphocyte count.

To study the prognostic significance of the AMC and ALC, both counts were obtained by complete blood count with differential (CBCD) at the time of diagnosis. There was a statistically significant but moderate association between ALC and prognosis (*p*-value = 0.0163, Cramer’s V = 0.371). Patients with ALC < 1.47 × 109/L had a worse outcome than those with ALC ≥ 1.47 × 109/L (30% of patients with low ALC had a good outcome vs. 69.2% of patients with high ALC had a good outcome) (Table 4, Figure 4).

**Table 4:** Patients’ distribution according to ALC cutoff and outcome. ALC: absolute lymphocyte count.

	ALC < 1.47 × 10^9^/L	ALC > 1.47 × 10^9^/L	Total	*p-*value
Poor outcome	11	8	19	0.0163
Good outcome	5	18	23	
Total	16	26	42	

**Figure 5:**
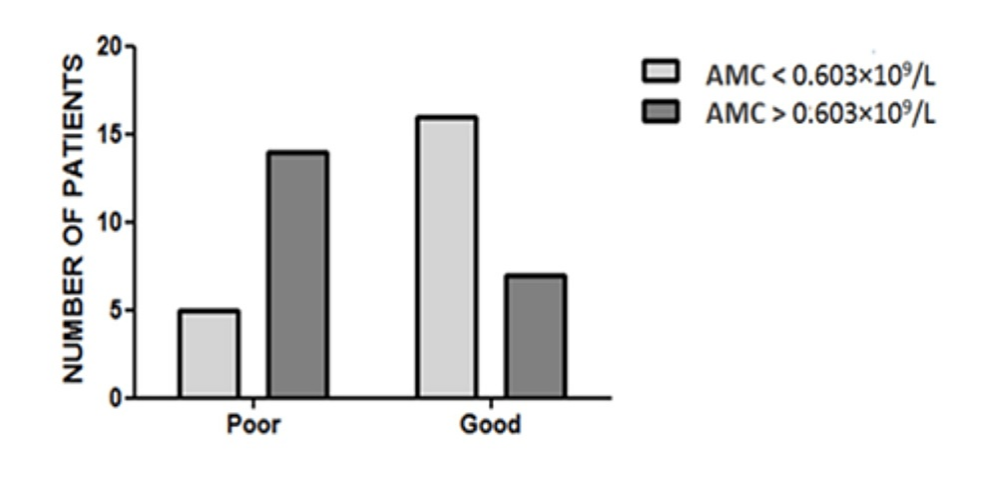
Patients’ distribution according to AMC cutoff and outcome. Significant moderately strong association between AMC and prognosis (*p* = 0.0053, Cramer’s V = 0.431). AMC: absolute monocyte count.

Patients with AMC < 0.603×109/L had a better outcome than those with AMC ≥ 0.603 × 109/L (76.2% of patients with low AMC had a good outcome vs. 33% of patients with high AMC had a good outcome). The association between AMC and prognosis was significant moderately strong (*p* = 0.0053, Cramer’s V = 0.431) (Table 5, Figure 5).

**Table 5:** Patients’ distribution according to AMC cutoff and outcome. AMC: absolute monocyte count.

	AMC < 0.603 × 10^9^/L	AMC > 0.603 × 10^9^/L	Total	*p-*value
Poor outcome	5	14	19	0.0053
Good outcome	16	7	23	
Total	21	21	42	

**Figure 6:**
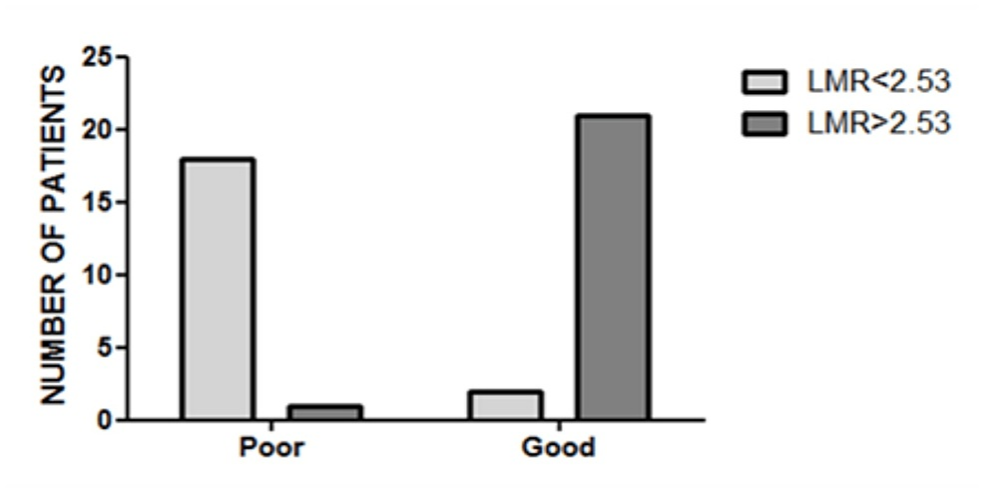
Patients’ distribution according to LMR cutoff and outcome. Very significant strong association between LMR and prognosis (*p* < 0.0001, Cramer’s V = 0.853). LMR: lymphocyte-to-monocyte ratio.

Patients with LMR < 2.53 had a significantly worse outcome than those with LMR ≥ 2.53 (10% of patients with LMR below the cutoff had a good outcome vs. 95.5% of patients with LMR above the cutoff had a good outcome). A very significant strong association was observed between LMR and prognosis (*p* <0.0001, Cramer’s V = 0.853) (Table 6, Figure 6).

**Table 6:** Patients’ distribution according to LMR cutoff and outcome. LMR: lymphocyte-to-monocyte ratio.

	LMR < 2.53	LMR > 2.53	Total	*p-*value
Poor outcome	18	1	19	<0.0001
Good outcome	2	21	23	
Total	20	22	42	

The association between LMR and R-IPI

Since R-IPI is the main prognostic factor in patients with DLBCL treated with R-CHOP [1], we compared the LMR with the R-IPI to determine whether LMR can further categorize patients within each R-IPI group. We combined the very good and good R-IPI risk groups (score 0-2). Within this group, very significant association between LMR and outcome was observed. Those patients were predicted to have a good response according to R-IPI, but 33% (nine of 27 patients) had a poor outcome. All of these nine patients (100%) with a poor outcome had LMR < 2.53. The results showed that within this low risk category, patients with LMR < 2.53 had a worse outcome than patients with LMR > 2.53 (82% of patients with LMR < 2.53 had a poor outcome vs. 0% of patients with LMR > 2.53 had a poor outcome with *p-*value <0.0001) (Table 7).

**Table 7:** Distribution of patients with good and very good R-IPI scores according to LMR and outcome. Very significant association between LMR and outcome was observed in patients with very good and good R-IPI scores (*p-*value <0.0001). LMR: lymphocyte-to-monocyte ratio; R-IPI: revised International Prognostic Index.

	LMR < 2.53	LMR > 2.53	Total	*p-*value
Good outcome	2	16	18	<0.0001
Poor outcome	9	0	9	
Total	11	16	27	

Among patients with poor R-IPI score, all of those who had a good outcome had LMR > 2.53. The results showed that patients in this category with LMR > 2.53 had a better outcome than patients with LMR < 2.53 (83% of patients with LMR >2.53 had a good outcome versus 0% of patients with LMR < 2.53 had a good outcome with *p-*value = 0.002) (Table 8).

**Table 8:** Distribution of patients with poor R-IPI score according to LMR and outcome. Significant association between LMR and outcome in patients with poor R-IPI scores (*p* = 0.002). LMR: lymphocyte-to-monocyte ratio; R-IPI: revised International Prognostic Index.

	LMR < 2.53	LMR > 2.53	Total	*p-*value
Good outcome	0	5	5	0.002
Poor outcome	9	1	10	
Total	9	6	15	

We also studied whether there is some association between LMR and R-IPI risk groups but no statistically significant association was found (*p-*value = 0.2492). The association was also not found between each of ALC and AMC and R-IPI (*p* = 0.55 and 0.189, respectively).

The association between LMR and gender

**Figure 7:**
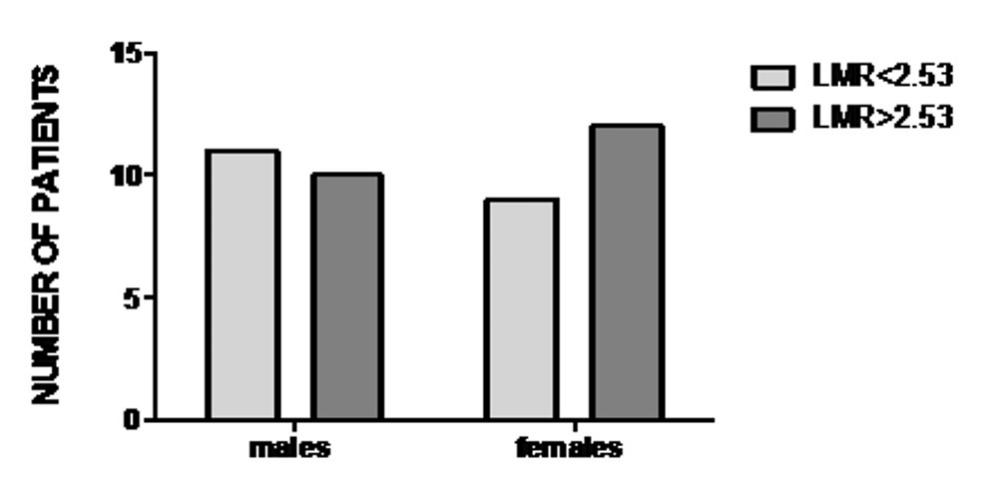
Distribution of patients according to LMR and gender. No significant association between LMR and gender (*p* = 0.536). LMR: lymphocyte-to-monocyte ratio.

No significant association was found between LMR and gender. Among patients with LMR < 2.53, 55% were males, while 45% were females. Among patients with LMR > 2.53, 45% were males, while 55% were females (*p *= 0.536) (Figure 7).

## Discussion

Many studies based on GEP and immunohistochemistry in NHL show that lymphocytes and myeloid cells in the tumor microenvironment influence the prognosis. Indeed, immunity and tumor microenvironment have an important role in the pathogenesis of lymphoma, and markers that consider these factors can provide more prognostic information than the available ones [15,16].

We tried to show the effect of peripheral lymphocyte and monocyte counts, as markers of patients’ immunity and tumor microenvironment, on patients’ outcomes. In this study, we also tried to confirm that LMR is important and informative in predicting the patients’ response to R-CHOP. We used ROC curve analysis to obtain cutoff values of LMR, ALC, and AMC.

Our results show that a decreased ALC and an elevated AMC with a decreased LMR negatively impact the prognosis. In fact, LMR was found to be a strong prognostic factor in patients treated with R-CHOP. LMR, at the defined cutoff, was also able to stratify patients within the R-IPI categories into low and high risk, thus predicting the high-risk patients in the low-risk R-IPI group and low-risk patients in the high-risk R-IPI group. No correlation was established between LMR and R-IPI score. This suggests that LMR may provide IPI-independent prognostic information that helps in the better prediction of patients’ prognosis.

In DLBCL, the elevated number of CD4+ cells in the tumor environment predicts a better outcome [16]. In addition, lymphopenia has been shown to play a role in poorly influencing the prognosis of patients diagnosed with Hodgkin’s lymphoma, DLBCL, and other subtypes of NHL [17,18]. Our study showed a similar association between ALC at admission and prognosis in DLBCL patients. Indeed, lymphocyte count is a surrogate marker of host immune competence. Immune suppression has been known to promote the development of lymphomas, the best example of which is lymphoproliferative disease post-transplantation [19]. In addition, it is the host immunity that mediates the therapeutic effect of rituximab [20].

Different mechanisms may be involved, including signaling-induced programmed cell death, complement-mediated cytotoxicity, and antibody-dependent cellular cytotoxicity (ADCC). By reducing the circulating lymphocytes, the effector cells are decreased, thus impacting ADCC. In addition, long-term cell-mediated immunity provided by presenting tumor antigens to T lymphocytes by dendritic cells will be affected by lymphopenia [20,21].

Baseline AMC was shown to adversely impact the survival of patients with DLBCL, HL, and follicular lymphoma (FL) [17,18]. Our study also suggests that elevated AMC at diagnosis has a poor outcome in DLBCL patients treated with R-CHOP therapy. The prognostic significance of an increased monocyte count in DLBCL may be explained by several mechanisms. A recent gene expression study showed that myeloid lineage cells infiltrating the tumor, including tumor-associated macrophages, increase its aggressiveness by secreting matrix metalloproteinase and inhibiting T-cell-mediated immunity [16]. Also, macrophages produce vascular endothelial growth factor A, promoting tumor angiogenesis [22]. In addition, monocytes are a source of B-lymphocyte stimulator (BLyS) that supports the growth of malignant B cells. Elevated BLyS was associated with poor survival and a progressive course of B-cell NHLs with a decrease in response to treatment [23]. Monocytes have also been shown to support the survival of lymphoma cells in T-cell and B-cell lymphoproliferative disorders [24].

In our study, we established cutoffs of ALC (1.47 ×109/L), AMC (0.603 ×109/L), and LMR (2.53) based on ROC curve to describe the prognostic impact of LMR on patients treated with rituximab. 95.5% of patients with LMR above 2.53 had a sustained response to Rituximab and no relapse during the follow-up period. 90% of patients with LMR < 2.53 had either not responded to treatment at all or relapsed during follow-up. 70% of patients with lymphocyte count below the cutoff had no response or early relapse, while 69.2% of patients with ALC above the cutoff had a sustained response. The prognostic impact of AMC was also significant with 76.2% of patients below the cutoff had a sustained response to R-CHOP and 67% of patients with AMC above the cutoff had no response or relapsed after treatment. There was no association between LMR and R-IPI, but the ability of LMR to stratify all R-IPI category patients into low and high risk was statistically significant. No association was noted between LMR and gender. These results were in concordance with the previously published studies in this domain.

In 2011, Wilcox et al. performed a retrospective study on 366 DLBCL patients. The established cutoffs of ALC and AMC were 1470/μL and 610/μL, respectively. ALC below the cutoff was associated with adverse outcome with estimated five-year OS and progression-free survival (PFS) of 53% and 43%, respectively. AMC above the cutoff was also associated with adverse outcome with estimated five-year OS of 49% and five-year PFS of 50% [18].

Another retrospective study done by Li et al. on 438 DLBCL patients showed that patients with ALC > 1.10 × 109/L seemed to have significantly better OS and PFS compared to patients with ALC ≤ 1.10 × 109/L. Patients with AMC ≥ 0.62 × 109/L had worse PFS and OS than patients with AMC < 0.62 × 109/L. Low LMR (cutoff established to be 2.6) at diagnosis predicted worse survival in DLBCL (five-year OS 70% ) compared to high LMR (five-year OS >80%). It could also identify patients with worse outcome within the low-risk IPI group. In addition, LMR < 2.6 was linked to unfavorable clinical characteristics, including poor performance status, elevated LDH level, and advanced stages [25].

Rambaldi et al. conducted a similar study on 1057 DLBCL patients at four centers that showed the prognostic significance of LMR in R-CHOP-treated patients independent of IPI score. LMR < 2.6 was associated with poor treatment response and worse survival (73% OS at four years) compared to LMR > 2.6 (86% OS at four years) [26].

A recent retrospective study done on a smaller population (69 patients) by Huang et al. concluded that only ALC and LMR at diagnosis were independent prognostic factors for OS and PFS at cutoffs of 1.0 × 109/L and 2.6, respectively. AMC did not show any prognostic significance at an established cutoff of 0.3 × 109/L [27].

A meta-analysis conducted by Xia et al. on 5012 patients with DLBCL from 12 reports to evaluate the prognostic role of LMR. It demonstrated that DLBCL patients with LMR < 3 had worse PFS (HR = 2.21, 95% CI = 1.82-2.73) and OS (HR = 1.75, 95% CI = 1.36-2.22) than patients with LMR > 3. This meta-analysis also performed stratification by cutoff, sample size, and country, and the prognostic impact of LMR was not changed [28].

Given the requirement to better predict patients who will not respond to R-CHOP, many predictors were studied. Some of the study parameters introduced prior to the rituximab era were found to be no more predictive after the introduction of rituximab to chemotherapy. For example, a study conducted by Gutiérrez-García et al. concluded that germinal versus non-germinal center phenotypes defined by immunohistochemistry are no more predictive for outcome in the rituximab era [13]. Another study done by Mounier et al. shows that rituximab eliminated the adverse prognostic role of Bcl-2 protein overexpression in DLBCL [11]. The gene expression profile revealed a prognostic significance, but it was not cost-effective, and the study was difficult to conduct [16,29]. By conducting many studies, the role of the tumor microenvironment in tumorigenesis was emphasized. However, these studies were based on immunohistochemistry and gene profiling [30]. In fact, including these data about the tumor microenvironment into the prognostic evaluation was difficult and not feasible. That is the main reason for performing many studies that focus on the relationship between lymphocyte and monocyte counts and prognosis. Indeed, these values are easily obtained by CBCD, and LMR can be easily calculated and included in practice. In addition, prognostic data provided by LMR are independent of R-IPI score and improve the risk definition by R-IPI by further stratifying patients within each R-IPI category.

Study limitations and perspective

Several limitations should be pointed out in our study. First, the sample size at our hospital was small. Many patients did not return for follow-up as recommended by their treating physicians, and some patients with DLBCL did not receive rituximab due to financial reasons. Second, the study was conducted at a single center. Third, we studied patients only in Lebanon, which has a small population size. Due to these limitations, the sample that we ended up studying was 42 patients.

The results of the study may pave the way for introducing new prognostic markers for DLBCL that should be considered when generating new prognostic scores. This could help physicians to better predict patients’ outcome and consider alternative therapy to be given to patients who would likely not achieve complete remission with R-CHOP. What is important is that these markers are easy to obtain and cost-effective. In addition, our results could improve the prognostic role of the current clinical prognostic index (R-IPI). Our study could have been more precise if we had included other centers, as these would have increased the sample size. However, the similarities between our results and other studies, including both smaller and larger sample size and meta-analysis, support our findings.

## Conclusions

Among the three parameters studied, LMR is a strong predictive and prognostic index for response in DLBCL patients treated with R-CHOP. This seems independent of the R-IPI commonly used. Despite the limited number of patients collected and the single center in which our study was conducted, the results obtained showed the importance of the association between lymphocyte and monocyte counts and patients’ prognosis. The strongest association was noted between the LMR and prognosis with a cutoff value of LMR of 2.53, which was close to the cutoff stated in the literature. For confirmation, this conclusion should be validated on another wider sample enrolling a larger number of patients at different centers.
